# Supplementary material for: Comparing the Informative Value of 2-Minute Segments of the 6-Minute Walk Test: Insights into a Prospective Study on Parkinson’s Disease
Source: Sensors (Basel). 2025 Nov 20;25(22):7076. doi: 10.3390/s25227076 (PMC12656093; doi:10.3390/s25227076)
Supplement: Supplementary file 1 [file sensors-25-07076-s001.zip › sensors-3919514-supplementary.pdf]

Table S1. Demographic, Clinical, and Motor Function Characteristics of the Study Subset vs. Original Cohort

(Variables	Study Subset (n=43)	Original Cohort (n=62)	*p*-value
Demographic and Clinical Characteristics			
Age (y), *median (IQR)*	65.0 (60.0- 69.5)	66.0 (60.3- 69.8)	
Sex, (% male)/ female	28 (65.1)/ 15	44 (71.0)/ 18	
Height (m), *median (IQR)*	170.0 (163.5- 178.0)	169.0 (163.2- 177.5)	
Weight (kg), *median (IQR)*	72.0 (62.5- 82.0)	77.50 (63.3- 82.8)	
BMI (kg/m^2), *median (IQR)*	24.8 (23.1- 27.0)	25.1 (23.5- 27.8)	
Hoehn and Yahr stage, *median (IQR)*	2.0 (2.0- 2.0)	2.0 (2.0- 2.0)	
Disease duration (y), *median (IQR)*	4.3 (2.1- 8.0)	3.95 (1.7- 6.9)	
Age at diagnosis (y), *median (IQR)*	61.2 (56.0- 64.0)	61.9 (57.2- 64.8)	
Age of onset (y), *median (IQR)*	59.4 (55.2- 63.2)	60.8 (55.9- 64.1)	
Subtype (AR/TD, %)	25 (58.1)/18 (41.9)	32 (51.6)/ 30 (48.4)	
Education level (<12 y), *n (%)*	26 (64.5)	40 (64.5)	
LEDD (mg), *median (IQR)*	450.0 (320.0- 730.0)	420.0 (300.0- 616.2)	
LED (mg), *median (IQR)*	300.0 (250.0- 462.5)	300.0 (162.5- 400.0)	
Motor function and Cognitive Assessments			
UPDRS III, *median (IQR)*	22.0 (18.0- 28.5)	19.5 (15.0- 25.0)	
Adjusted MMSE, *median (IQR)*[1,2]	28.0 (27.0- 29.1)	28.0 (27.0- 29.2)	
Adjusted MoCA, *median (IQR)*[1]	27.0 (23.5- 28.0)	26.0 (23.3- 28.0)	
IPAQ (Low/Mod/High)[3], *n (%)*	9 (20.9)/ 23 (53.5)/ 11(25.6)	19 (30.7)/ 30 (48.4)/ 13 (20.9)	<0.001

Study Subset (Center BG and MB), Original Cohort (Center BG, MB, BS). BMI, Body Mass Index. HY stage, Hoehn and Yahr stage. AR, Akinetic-Rigid; TD, Tremor-Dominant. Levodopa Equivalent Daily Dose – LEDD is the amount of the daily dopaminergic drugs. L-Dopa Equivalent Dose – LED is the amount of the daily L-Dopa intake. UPDRS, Unified Parkinson's Disease Rating Scale (Part III- Motor Exam). MMSE, Mini–Mental State Examination. MoCA, Montreal Cognitive Assessment [Adjustment for [1]education level and [2]age]. [3]IPAQ, International Physical Activity Questionnaire classification. Continuous variables are expressed as median and interquartile range (IQR), categorical ones as count and percentage. Only p-value <0.05 are reported.

The comparison between the study subset and the original cohort revealed no significant differences in demographic and clinical characteristics, or cognitive assessment. However, a significant difference was observed in the distribution of physical activity levels, as assessed by the IPAQ, with the study subset showing a higher proportion of participants with moderate physical activity compared to the original cohort. The overall incidence (original cohort) of falls in the month preceding the motor assessment was low (4.84%), with three patients reporting one event, and only one participant experienced a fall during the ON phase. Notably, motor performance (UPDRS III) and dopaminergic treatment (LEDD and LED) were comparable between the two groups, ensuring a consistent motor profile.

Comparison of Demographic Characteristics and Biomechanical Parameters Between Centers

The time for the 6MWT was standardized at 6 minutes for all participants, although a slight difference was noted (6.04 vs. 6.0 minutes), which may reflect variations in timing procedures. Stride length exhibited modest differences, but the symmetry index remained consistent, indicating similar gait mechanics and limb coordination between patients at both centers. Gait speed, cadence, and distance covered depend on both stride length and cadence.

The symmetry index (General Symmetry Index, GSI), as calculated by the G-Walk, reflects the relative difference in stance or swing phases between the left and right limbs, providing a measure of inter-limb coordination. The GSI is computed over the entire walking trial, and extracting shorter subsegments, such as the first 2 minutes, would have required reprocessing the data, which could potentially

affect the accuracy of the gait parameters. Due to limitations in re-sampling the raw G-Walk data, symmetry values for the first 2 minutes could not be reliably calculated to match those derived from the full 6MWT. Therefore, these data were omitted from the current analysis. When stride length is consistent, differences in gait speed and distance are likely driven by variations in cadence. Age, height, weight, and disease stage were carefully controlled and found to be comparable between the two centers. Potential differences in sensor calibration should also be considered when interpreting the biomechanical differences observed. Differences emerged in physical activity levels and dopaminergic treatment protocols, although the use of adjunctive therapies (e.g., dopamine agonists, MAO-B inhibitors, COMT inhibitors) remained consistent between centers (data not shown, LEDD-LED). A significant difference in disease duration was observed, with patients from BG Center showing a longer duration compared to those from MB Center. At BG Center, diagnosis occurred immediately after the onset of motor symptoms (0 years from onset to diagnosis), while at MB Center, there was an approximate 1-year delay. Nonetheless, no differences were identified in age at disease onset, age at diagnosis, age at therapy initiation, or the actual age of participants. Other demographic and clinical characteristics were comparable, supporting the validity of comparisons between the two centers.

Table S2. Demographic and Clinical Characteristics of Participants in the Study subset at Basal Evaluation, comparison between BG and MB centers

	**Center BG**	**Center MB**	***p*-value**
N=43	24	19	
Age (y), *median (IQR)*	65.0 (60.8- 69.2)	65.0 (60.0- 70.0)	
Sex, (% male)/ female	15 (62.5)/ 9	13 (68.4)/ 6	
Height (m), *median (IQR)*	169.0 (165.0- 175.0)	171.0 (162.0- 178.0)	
Weight (kg), *median (IQR)*	71.0 (61.2- 78.5)	75.0 (64.0- 85.0)	
BMI (kg/m^2), *median (IQR)*	24.3 (21.3- 26.4)	25.7 (23.6- 28.3)	
Hoehn and Yahr stage, *median (IQR)*	2.0 (2.0- 2.0)	2.0 (2.0- 2.0)	
Disease duration (y), *median (IQR)*	6.2 (3.9- 9.0)	2.7 (1.05- 4.3)	0.017
Age at diagnosis (y), *median (IQR)*	57.6 (54.2- 64.7)	62.3 (58.9- 63.8)	
Age of onset (y), *median (IQR)*	57.6 (54.2- 64.0)	60.4 (57.8- 62.4)	
Subtype (AR/TD), *n (%)*	14 (58.3)/10 (41.7)	12 (63.2)/7 (36.8)	
UPDRS III, *median (IQR)*	22.5 (17.8- 32.0)	22.0 (18.5- 24.0)	
Adjusted MMSE, *median (IQR)*[1,2]	28.0 (27.4- 30.0)	27.4 (25.8- 29.0)	
Adjusted MoCA, *median (IQR)*[1]	27.0 (25.5- 28.0)	26.0 (22.5- 28.0)	
IPAQ (Low/Mod/High)[3], *n (%)*	8(33.3)/ 15(62.5)/ 1(4.2)	1(5.3)/ 8(42.1)/ 10(52.6)	<0.001
Therapies, median (IQR)			
LEDD (mg)	555.0 (400.0- 790.0)	352.0 (228.0- 507.0)	0.014
LED (mg)	325.0 (300.0- 600.0)	300.0 (100.0- 300.0)	0.026
6MWT, median (IQR)			
Duration (min)	6.0 (6.0- 6.0)	6.0 (6.0- 6.6)	<0.001
Distance covered (m)	358.0 (334.0- 378.0)	304.0 (286.0-330.0)	<0.001
Stride length (m)	1.4 (1.3- 1.5)	1.3 (1.1- 1.4)	0.032
Gait Speed (m/s)	1.4 (1.3- 1.5)	1.1 (1.0- 1.2)	<0.001
Symmetry Index	96.5 (92.4- 97.6)	93.1 (93.1- 96.6)	
Cadence (step/min)	119.0 (115.0- 123.0)	109.0 (103.0- 114.0)	<0.001

2'6MWT, median (IQR)			
Duration (min)	2.0 (1.9- 2.0)	2.0 (2.0- 2.1)	
Distance covered (m)	120.0 (118.0- 129.0)	96.0 (96.0- 115.0)	0.002
Stride length (m)	1.4 (1.3- 1.5)	1.3 (1.1- 1.4)	
Gait Speed (m/s)	1.4 (1.3- 1.5)	1.1 (1.0- 1.2)	<0.001
Cadence (step/min)	121.0 (118.0- 126.0)	110.0 (104.0- 116.0)	<0.001

Study Subset (Center BG and MB). BMI, Body Mass Index. HY stage, Hoehn and Yahr stage. AR, Akinetic-Rigid; TD, Tremor-Dominant. UPDRS, Unified Parkinson's Disease Rating Scale (Part III- Motor Exam). MMSE, Mini–Mental State Examination. MoCA, Montreal Cognitive Assessment [Adjustment for [1]education level and [2]age]. [3]IPAQ, International Physical Activity Questionnaire classification. Levodopa Equivalent Daily Dose – LEDD is the amount of the daily dopaminergic drugs. L-Dopa Equivalent Dose – LED is the amount of the daily L-Dopa intake. Continuous variables are expressed as median and interquartile range (IQR); categorical ones as count and percentage. Only p-value <0.05 are reported.

Table S3. Sensitivity analysis: Passing–Bablok regression (compared with standard linear regression)

Test parameter	*Linear Regression*		*Passing–Bablok regression*	
	Slope (95% CI)	*Intercept*	*Slope (95% CI)*	*Intercept*
Distance covered (m)	2.68 (2.30- 3.05)	31.33	2.90 (2.64- 3.24)	10.08
Stride length (m)	**1.0 (0.96- 1.06)**	**-0.01**	**1.0 (0.95- 1.07)**	**0.0**
Gait Speed (m/s)	**0.99 (0.93- 1.05)**	**-0.0**	**1.02 (0.94- 1.08)**	**-0.04**
Cadence (step/min)	0.94 (0.90- 0.98)	5.66	0.97 (0.93- 1.04)	1.76

Interpretation: slope >1 strong relationship, slope= 1 and intercept =0 perfect linear relationship. N= 43, missing: 1 unable to complete the 6 minutes of the test. Bold indicates gait parameters showing consistent results between the two analyses.

Analysis of 2-Minute Segments of the 6MWT

To explore potential changes in gait performance during the 6MWT, the data were divided into three consecutive 2-minute segments. Raw G-Walk data were segmented cumulatively by summing consecutive patches (each representing two steps of the same foot) until reaching approximately 2 minutes per segment. This procedure ensured that each segment represented an actual 2-minute duration, independent of the patient's gait speed. Accordingly, Segment 1 corresponded to the first 2 minutes, Segment 2 to the following 2 minutes, and Segment 3 to the final 2 minutes of the test.

This segmentation allowed a preliminary examination of differences between the initial and final phases, providing insight into possible gait fatigability during the test. The symmetry index was not calculated for partial 2-minute segments, as it cannot be reliably estimated, and was therefore excluded from this analysis.

Table S4. Comparisons across three 2-minute segments of the 6MWT

**Variables**	**Comparison**	**Δ**	**Effect size**	**p (adj.)**
Duration (min)	*All comparisons*	~ 0	r <0.1	ns
Distance covered (m)	*Segm 1- Segm 2*	0 (-9.6, 0)	**r = 0.33**	**0.003**
	Segm 1- Segm 3	–1.55 (-9.9, 0)	**r = 0.28**	**0.014**
	Segm2- Segm 3	0 (-1.58, 0)	r = 0.05	ns
Stride length (m)	*All comparisons*	~ 0	r <0.1	ns
Gait Speed (m/s)	*All comparisons*	~ 0	r <0.1	ns

Cadence (step/min)	*Segm 1- Segm 2*	-1.98 (-3.07, -0.65)	**r = 0.41**	**<0.001**
	Segm 1- Segm 3	-2.12 (-3.46, 0.07)	**r = 0.32**	**0.008**
	Segm2- Segm 3	-0.16 (-1.05, 0.66)	r = 0.08	ns

Results of repeated-measures Friedman tests (global effect) and Bonferroni-adjusted Wilcoxon pairwise comparisons across three consecutive 2-minute segments (Segm 1, Segm 2, Segm 3) of the 6MWT. For each gait variable, differences (Δ median (IQR)) between segments are reported, along with adjusted p values and effect sizes (r, interpretation: ~ 0.1 small, ~ 0.3 moderate, and ≥0.5 large) for pairwise comparisons. ns: non-significant comparisons ($p > 0.05$). "All comparisons" refers to the overall Friedman test (global effect) across segments when the global effect was not significant. Significant results marked in bold (with p-value < 0.05). The analysis includes data from 42 patients who completed the 6MWT (1 patient was unable to complete the full 6 minutes).

Table S4 shows the comparison between the first 2 minutes of the 6MWT with subsequent 2-minute segments. The results indicated that patients start the 6MWT with a more vigorous gait pattern, covered a slightly greater distance and presented higher cadence in the initial segment, followed by a modest reduction in distance and cadence during the later segments, while gait speed and stride length remained stable (Friedman test: distance $\chi^2 = 11.9$, $p = 0.003$, Kendall's $W = 0.14$; cadence $\chi^2 = 23.7$, $p < 0.001$, $W = 0.28$; gait speed, stride length, and duration: ns). Corresponding effect sizes indicated small-to-moderate changes (r = 0.28–0.41). These findings suggest that the observed reduction is mainly driven by a decrease in step frequency rather than changes in step amplitude or overall walking rhythm.

Overall, the first 2 minutes provide a reasonable approximation of total walking capacity, supporting the notion that a 2-minute walk test may serve as a practical surrogate for the full 6MWT when time or patient tolerance is limited. However, only the complete 6-minute test captures the subtle, clinically relevant changes associated with fatigability.

Table S5: Comparative Analysis of Gait Parameters in the First 2 Minutes vs. Total 6-Minute Walk Test. 1- year Follow-up

Tests	2 minutes	6 minutes	*Paired Test*	*Spearman Correlation*	*Linear Regression*	
6MWT			*p-value**	*r (p-value)*	*Slope (95% CI)*	*Intercept*
Distance covered (m)	111.8 (96.0- 121.3)	335.4.7- 366.4)	**-**	**0.907 (<0.001)**	2.86 (2.42- 3.29)	13.90
Stride length (m)	1.3 (1.2- 1.5)	1.3 (1.2- 1.5)	ns	**0.931 (<0.001)**	0.84 (0.74-0.95)	0.18
Gait Speed (m/s)	1.3 (1.1- 1.5)	1.3 (1.1- 1.4)	**<0.001**	**0.971 (<0.001)**	**0.91 (0.83-1.0)**	**0.07**
Cadence (step/min)	118.6 (108.3- 124.4)	116.8(109.0- 122.7)	**<0.001**	**0.897 (<0.001)**	0.78 (0.66- 0.91)	23.94

The data of 2 and 6 minutes of 6MWT are given as median values with interquartile range (IQR). Significant results (p-value < 0.05) marked in bold. ns: non-significant. *Paired comparisons were performed using the Wilcoxon signed-rank or Sign test. Interpretation: r > ±0.7: strong correlation; slope >1 strong relationship, slope= 1 and intercept =0 perfect linear relationship. N= 40, missing: 2 patients deceased, 1 patient unable to complete the 6 minutes of the test. Bold indicates parameters for which the 2-minute and 6-minute measurements showed a consistent relationship, being statistically significant in all three analyses

Table S6: Concordance and Reliability of Gait Parameters in the First 2 Minutes vs. Total 6-Minute Walk Test. 1- year Follow-up

Gait parameter	***CCC (95% CI)***	***Bland-Altman***
		Average difference (95%CI of LoA)
Distance covered (m)	0.04 (-0.02- 0.05)	0.04 (0.02-0.05)
Stride length (m)	**0.93 (0.87- 0.96)**	**-0.02 (-0.19- 0.14)**
Gait Speed (m/s)	**0.95 (0.90-0.97)**	**-0.04 (-0.16- 0.08)**
Cadence (step/min)	0.89 (0.79- 0.93)	-0.85 (-9.78- 8.11)

CCC, Concordance Correlation Coefficient and corresponding 95% confidence interval (CI). LoA, Limits of Agreement. Significant results marked in bold. Interpretation: CCC: 0-0.90: poor agreement; 0.91-0.99: almost perfect agreement. Bland–Altman average difference = 0 perfect agreement; (≠ 0) lack of agreement between the two variables. Missing: 2 patients deceased, 1 patient unable to complete the 6 minutes of the test. Bold indicates gait parameters showing consistent results between the two analyses.

Table S7: Comparative Analysis of Gait Parameters in the First 2 Minutes vs. Total 6-Minute Walk Test. 2- year Follow-up

**Tests**	2 minutes	6 minutes	***Paired Test***	***Spearman Correlation***	***Linear Regression***	
6MWT			*p-value**	*r (p-value)*	*Slope (95% CI)*	*Intercept*
Distance covered (m)	111.8 (96.0- 120.4)	342.0 (295.6- 359.3)	**-**	**0.832 (<0.001)**	1.0 (0.42- 1.59)	220.96
Stride length (m)	1.3 (1.1- 1.4)	1.3 (1.1- 1.4)	ns	**0.976 (<0.001)**	**0.96 (0.88- 1.03)**	**0.05**
Gait Speed (m/s)	1.3 (1.1- 1.4)	1.3 (1.0- 1.4)	ns	**0.953 (<0.001)**	**0.95 (0.86-1.04)**	**0.05**
Cadence (step/min)	120.0 (111.4- 127.0)	117.6 (112.2- 125.3)	**<0.001**	**0.980 (<0.001)**	0.93 (0.87- 1.0)	6.67

The data of 2 and 6 minutes of 6MWT are given as median values with interquartile range (IQR). Significant results (*p*-value < 0.05) marked in bold. ns: non-significant. *Paired comparisons were performed using the Wilcoxon signed-rank or Sign test. Interpretation: r > ±0.7: strong correlation; slope >1 strong relationship, slope= 1 and intercept =0 perfect linear relationship. N= 35, missing: 2 patients deceased, 3 patients unable to complete the 6 minutes of the test, 2 patients lost at follow-up, 1 patient dropped out. Bold indicates parameters for which the 2-minute and 6-minute measurements showed a consistent relationship, being statistically significant in all three analyses

Table S8: Concordance and Reliability of Gait Parameters in the First 2 Minutes vs. Total 6-Minute Walk Test. 2- year Follow-up

Gait parameter	***CCC (95% CI)***	***Bland-Altman***
		Average difference (95%CI of LoA)
Distance covered (m)	0.02 (0.01- 0.04)	0.04 (0.02-0.05)
Stride length (m)	**0.97 (0.95- 0.99)**	**0.0 (-0.08- 0.08)**
Gait Speed (m/s)	**0.97 (0.94-0.98)**	**-0.0 (-0.10- 0.10)**
Cadence (step/min)	**0.97 (0.94- 0.98)**	-1.28 (-4.66- 2.11)

CCC, Concordance Correlation Coefficient and corresponding 95% confidence interval (CI). LoA, Limits of Agreement. Significant results marked in bold. Interpretation: CCC: 0-0.90: poor agreement; 0.91-0.99: almost perfect agreement. Bland–Altman average difference = 0 perfect agreement; (≠ 0) lack of agreement between the two variables. Missing: 2 patients deceased, 3 patients unable to complete the 6 minutes of the test, 2 patients lost at follow-up, 1 patient dropped out. Bold indicates gait parameters showing consistent results between the two analyses.

Table S9. Clinical and treatment measures over time

Variables	Baseline (n=43)	1-year (n=42)	2-year (n=38)	p (adj.)
Demographic and Clinical Characteristics				
BMI (kg/m^2), *median (IQR)*	24.8 (23.1- 27.0)	24.8 (22.2- 26.6)	24.8 (22.2- 26.6)	ns
Hoehn and Yahr stage, *median (IQR)*	2.0 (2.0- 2.0)	2.0 (2.0- 2.0)	2.0 (2.0- 2.0)	ns
HY stage >2, *n (%)*	1(2.3)	2(4.8)	4(10.5)	-
LEDD (mg), *median (IQR)*	450.0 (320.0- 730.0)	315.0 (359.0- 685.0)	520.0 (408- 700)	
LED (mg), *median (IQR)*	300.0 (250.0- 462.5)	300.0 (300.0- 500.0)	375.0 (300.0- 506.2)	baseline vs 2-year: 0.002<br>1- vs 2-year: 0.041
Motor function and Cognitive Assessments				
UPDRS III, *median (IQR)*	22.0 (18.0- 28.5)	21.5 (15.0- 26.8)	19.5 (15.2- 25.8)	ns
Adjusted MMSE[1,2], *median (IQR)*[1,2]	28.0 (27.0- 29.1)	29.0 (27.0- 30.0)	29.0 (27.0- 30.0)	baseline vs 2-year: 0.048
Adjusted MoCA[1], *median (IQR)*[1]	27.0 (23.5- 28.0)	27.0 (25.0- 28.0)	26.5 (24.0- 27.0)	1- vs 2-year: 0.02

Results of repeated-measures Friedman tests and Bonferroni-adjusted Wilcoxon pairwise comparisons across time points. BMI, Body Mass Index. HY stage, Hoehn and Yahr stage. UPDRS, Unified Parkinson's Disease Rating Scale (Part III- Motor Exam, range, 0–132). MMSE, Mini–Mental State Examination. MoCA, Montreal Cognitive Assessment [Adjustment for [1]education level and [2]age]. Levodopa Equivalent Daily Dose – LEDD is the amount of the daily dopaminergic drugs. L-Dopa Equivalent Dose – LED is the amount of the daily L-Dopa intake. Continuous variables are expressed as median and interquartile range (IQR).

Table S9 provides an overview of the 2-year follow-up. Cognitive measures (MMSE and MoCA) remained stable throughout visits, with no significant differences observed. Motor scores also remained stable. In contrast, physical activity showed a progressive decline, with the proportion of participants in the low IPAQ category increasing from 20.9% to 42.1% (data not shown). Meanwhile, both LED and

LEDD increased significantly over time, reflecting adjustments in dopaminergic therapy, likely related to disease progression or management of motor symptoms.
